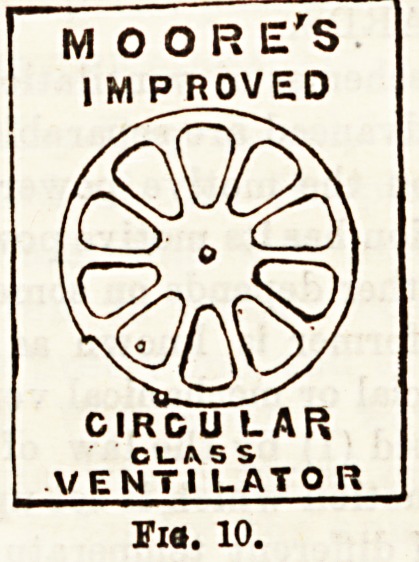# "The Hospital" Nursing Mirror

**Published:** 1896-05-16

**Authors:** 


"The Hospital" Nursing Mirror.

Being the Extra Nursing Supplement of "The Hospital" Newspaper.

[Contributions for this Supplement should be addressed to the Editor, The Hospital, 428, Strand, London, W.C., and should have the word "Nursing" plainly written in left-hand top corner of the envelope.]

### News from the Nursing World.

### DUKE AND DUCHESS OF FIFE AT BRIGHTON.

The Duke and Duchess of Fife on Tuesday laid the foundation-stone of a new nurses' home at the Royal Alexandra Hospital for Sick Children at Brighton. They were received on arrival at the hospital by Mr. W. L. Dewe (chairman of the board of management), Captain the Hon. R. Bingham (vice-chairman), Sir Joseph Ewart, and the Vicar of Brighton. After the ceremony, which was preceded by a short service of dedication, the Duchess was presented with a number of purses in aid of the building fund.

### PROGRESS AT QUEEN CHARLOTTE'S.

The uniform worn by the nurses and midwives at Queen Charlotte's Hospital is of white piqué, and with the addition of large white linen aprons, and neat muslin caps, has a very clean and business-like effect. Cleanliness, indeed, surgically speaking, as well as that which appeals to the eye, is the order of the day throughout the hospital. Recently the shortest period for which monthly nurses are trained has been increased to twelve weeks, and the desirability of devoting six months to learning such an important branch of nursing work is strongly impressed upon her pupils by Miss McCord, the matron. Unfortunately the question of fees prevents too many from following so wise a counsel. The nursing arrangements at Queen Charlotte's have often been thought to be somewhat behind the times, but under Miss McCord's rule an improved condition of affairs seem to prevail. Notably we believe the very reprehensible custom of allowing the pupil nurses to sleep *in* the wards has been abandoned, and a proper system of night nursing established.

### ACTION FOR RECOVERY OF DIPLOMA.

An action has been lately brought by a nurse, Sarah Jane Noble, at Northallerton, against Miss G. Atkinson, lady superintendent of the Northallerton Cottage Hospital and North Riding Rural Nursing Association, to recover a "nursing diploma," alleged to be wrongfully withheld, or "£1, its value, and £2 damages for detention." The plaintiff, in September, 1893, signed an engagement with the North Riding Association for five years. Part of her training she received at the Northallerton Hospital and part at the Newcastle Infirmary, also going for three months to the Glasgow Maternity Hospital, where she passed an examination and received a certificate, which certificate, on her return to Northallerton, was handed by her to Miss Atkinson. Nurse Noble was dismissed on December 26th, 1895 (according to the annual report of the hospital, for "disobedience and conduct unbecoming her profession"), and Miss Atkinson informed her that her diploma would be retained unless she paid £15, the cost of her training at Glasgow. Judge Turner held that a diploma was not a testimonial, and, therefore, the rule that testimonials were forfeited on dismissal did not apply; but although grave misconduct did not entitle the defendant to detain the diploma, as the plaintiff had bound herself for five years to the association the committee were entitled to hold the diploma until the expiration of that term in 1898. He, therefore, gave a verdict for the defendant, with costs.

### COMMISSIONS FOR NURSES.

An interesting discussion has been carried on lately in *Nursing Notes* anent "Nurses' Commissions," from which it is evident that the practice of offering commission on orders received through nurses on the part of chemists, instrument makers, and others is very prevalent. This is, of course, quite a different thing to nurses taking "professional discount" on goods ordered for their own use, a perfectly allowable practice. The general feeling elicited seems to be that the "whole system is unprofessional," and that nurses who respect themselves and their calling will do well not to give in to a bad custom, one which in the medical profession is looked upon with deep disapproval by the best class of practitioners. Another correspondent, who rightly considers that "the patients should have the benefit of any discount a nurse is able to obtain on instruments, dressings, appliances, &c.," also touches on the vexed question of private nurses' "tips," truly remarking that "the fear that tips will be expected adds to the dislike so constantly expressed against employing a trained nurse." The practice of accepting presents from patients seems to be growing, and, speaking broadly, it is an evil one, which nurses who care for the honour of their profession will do well to combat.

### RATE-SUPPORTED HOSPITALS.

Residents in the poorer parts of London are sometimes credited with a desire to substitute rate-supported hospitals for the voluntary ones. What a rate-supported hospital, with its absence of publicity, may mean, may be gathered from the following extract from a petition to the Local Government Board by Miss Marian M. Evans, matron of the infirmary at Rotherhite, under the St. Olave's Board of Guardians, as given in our contemporary *London* for the 7th inst.: "It has not appeared to me advisable or conducive to efficiency or discipline that individual members of the Visiting Committee should take tea in a nurse's duty-room or keep her from her patients; or that members of the committee should be smoking in the nurse's sitting-room at midnight; or that young nurses and probationers should go to smoking concerts (even though guardians and officers of the infirmary were present) and remain out until after midnight; or that nurses should have a general pass to remain out until half-past eleven p.m." We refrain from comment until the public inquiry, which must no doubt follow, has been held.

### ON BEHALF OF THE BABIES.

A new organisation has been started in Bristol with the object of lessening the present high rate of infant mortality. The aim of the society, of which the director is Dr. W. L. Christie, is to induce women of education and leisure to study simple questions of hygiene and infant feeding, &c., and to undertake to visit among the poor and instruct mothers in the care of their little ones. That woeful ignorance in the simplest matters connected with feeding prevails among the poorer classes, no one will deny, but those same classes have a true British objection to interference, and the ladies of the association are likely to have a hard task before them if they aspire to shake the factory mothers of Bristol from their settled convictions of what babies should thrive upon. The association will do good work if its members are able to spread abroad even the most elementary knowledge of proper feeding, and the value of fresh air and soap and water.

### AN INTERESTING EXHIBITION.

During the present week a very interesting Amateur Art Exhibition and Loan Collection of pictures and curios, in aid of several charities, amongst others the East London Nursing Fund, is on view at Mr. Reuben Sassoon's house, 1, Belgrave Square. The Hon. Mrs. Lowther is acting as president, with a large staff of energetic helpers. Last year a similar exhibition resulted in the substantial sum of £500, and as on this occasion the pictures are particularly interesting, even better things may be expected. The collection of Edridge's portraits, amongst which are a number lent by the Queen from Windsor, is considered to be the best ever get together.

### NURSING AT LINLITHGOW.

The Linlithgow District Nursing Association reports a good year's work, and a satisfactory balance in hand. At the recent annual meeting the committee very wisely decided to alter one of their rules stating that the association should consist of all subscribers and "Protestant clergymen" of the district. It was felt that although the association was actually entirely unsectarian, and the nurse attended cases entirely regardless of religious distinction, the introduction of the phrase in question might lead to misconception on the point, and the words were therefore eliminated from the rule. An isolation hospital is greatly needed for Linlithgow, but hitherto it has proved impossible to stir up the local authority on the matter. It was suggested at the meeting that the Ladies' Committee should make a further endeavour to bring about its erection, in which we hope they may be successful.

### MEDICAL MISSIONS AT TRAVANCORE.

Miss MacDonnell, who is in charge of the nursing department of the South Travancore Medical Mission, is to be congratulated on having accomplished her desire of building a special maternity ward. Her energetic appeals met with so good a response from friends in India and Scotland that besides completing the ward, it has been possible to organise a new operation room and put up a "nurses' home" just behind the hospital building. The latter is only a good-sized native house, but it is airy and pleasant, and brightened as much as possible by the addition of pictures. Miss MacDonnell has a busy time training native women as nurses and midwives, and just now, with a view to their better instruction, has another scheme in hand, the publication of a Tamil translation of Haultain and Ferguson's "Text-book of Midwifery." The native nurses for the most part manifest much interest in their work, and take great pains to learn.

### A SUCCESSFUL ENTERTAINMENT.

The "Café Chantant and May Day Revel," held at Queen's Hall last week in aid of the Hospital for Incurable Children, Maida Vale, was well attended. The presence of many children in quaint costumes made the scene a particularly pretty one, and the maypole dances, arranged by Mrs. Wordsworth, were well worth seeing. The programme was a varied one, and well carried through, and, to judge by the number of smart people who found their way to the Queen's Hall on the occasion, the result should be a handsome addition to the funds of this deserving little institution. Princess Edward of Saxe-Weimar was present in the afternoon.

### PENZANCE NURSING ASSOCIATION.

The Penzance Nursing Association has ceased to exist. Subscriptions have fallen off and the committee have decided that to carry on the work under present circumstances would be impracticable. The sick poor of the district are not, however, to suffer from this unfortunate lack of support on the part of the inhabitants of Penzance, thanks to the generosity of Mr. and Mrs. T. R. Bolitho, who have undertaken to provide a permanent district nurse under the terms of the Emily Bolitho Endowment Fund. At first it was proposed to support the nurse from this source, and "to secure her a proper connection with the medical authorities by asking the Infirmary Committee to undertake the management"; but after some negotiation this plan has fallen through, and the scheme will now be carried out independently of the infirmary, the details yet remaining to be arranged.

### SHORT ITEMS.

The Midwives Registration Bill, down for second reading on May 6th, was not discussed, Government business taking precedence. There is apparently now little chance of the Bill coming before Parliament this session.—Certificates of merit and medals were presented to the nurses of the Hastings and St. Leonards Hospital the other day by Mrs. Lucas Shadwell, who is an energetic worker on behalf of the institution.—The Children's Association, in connection with the North-Eastern Hospital for Children, is about to issue a quarterly magazine, to be devoted to the interests of the children of the poor in the districts surrounding the hospital. The first number will contain portraits of the Duchess of Connaught's children and a variety of illustrated stories, amongst others a sketch by John Strange Winter, "Some Children I Know."—Nurse Clarke, of the Red Cross Society, was amongst the passengers who reached Buluwayo safely by coach on May 1st. The building used as the Stock Exchange is being converted into a temporary hospital for sick and wounded.—The Rochdale Nursing Association is a new venture, and was looked upon with little favour by some at its initiation. The first quarter's working, however, seems to have been highly satisfactory, and at a special meeting held the other day unanimous approval was expressed of the nurses and their labours amongst the poor.

### Hygiene: For Nurses.

By John Glaister, M.D., F.F.P.S.G., D.P.H.Camb., Professor of Forensic Medicine and Public Health, St. Mungo's College, Glasgow, &c.

### VI.—METHODS OF VENTILATION APPLIED TO ROOMS, LARGE BUILDINGS, AND HOSPITAL WARDS.

All schemes of ventilation that have yet been, or may yet be, advanced are separable into two main divisions, depending on the motive power which originate them. The one division has its motive power in the ordinary forces of nature, the other depends on some form of artificial mechanical aid. The former is known as natural ventilation, the latter as artificial or mechanical ventilation. Natural ventilation is effected (1) by the law of the diffusion of gases, and (2) by the motion which is set up between two columns or bodies of air of different temperatures and pressure. By the former law we find that gases of different densities (or weight) will eventually mix uniformly, even against the law of gravity; hence the uniformity of composition of the atmosphere; and practical evidence of the latter is afforded by the motion of the atmosphere which we call wind, or by the ascension of smoke in the chimney. Gases expand by heat and contract by cold, and the amount of expansion of a gas for every degree of increase of temperature is at once definite and determinable. For every increase of temperature of 1° Cent., a gas expands $\frac{1}{273}$ part of its volume, and for 1° Fahr. above 32°, $\frac{1}{491}$ part; or, expressed in decimal fractions, for every degree Cent. the volume of a gas will become 1 + ·00366, and for every degree Fahr. 1 + ·002. As a gas expands, its increase of bulk demands more space, and it causes the air which surrounds it to move. Varying degrees of pressure, too, cause air movements. When the wind blows across a chimney-top it causes a partial vacuum in the chimney shaft, and a consequent upward movement in the air of the lower part of the shaft. This action is well illustrated in the ordinary spray apparatus. The steam which issues from the horizontal tube creates a partial vacuum in the perpendicular tube, and causes the medicated fluid to rise and become mixed with the steam. This simple diagram Fig. 5 will illustrate the point.

Fig. 5 is a bent glass tube, containing a small ball of fine cotton-wool, F. If a current of air be blown over G, from A, or B, the ball will traverse the glass-tube between F and G; but if from C, or D, then from F to E.

Mechanical Ventilation. — All schemes of this class are divisible into two sub-divisions, viz., (1) those where the fresh or pure air is forced into a building under pressure—hence called the propulsion or *plenum* method—and (2) those where the foul air is drawn out—known as the extraction or *vacuum* method.

Fig. 6 illustrates the extraction method, as carried out in pits and mines. This apparatus may be made at a cost of a few pence. It consists of a cardboard, air-tight, box, and two glass funnels which communicate with the interior of the box by holes in the cardboard. At the bottom of the funnel on the right is a short candle, which, when lighted, causes a current of air to pass down through the left funnel, to traverse the box, and pass up the right funnel. If the funnels represented the two shafts of a pit, the box the underground workings, and the candle the furnace at the bottom of the ventilating shaft, the parallel is complete. The path of the air when the candle is lighted is better demonstrated by holding smoking brown paper over the opening of the left funnel.

The figure also exemplifies natural ventilation, and air motion induced by unequal temperatures. As the plenum

Fig. 6.—Ventilation in Mines.

and vacuum methods will be discussed later, the problem of ventilation of a sick room may now be considered.

In the average ordinary room the only available means of ventilation are (1) the door, (2) window, and (3) fireplace. With a fire in the room, the two former act as inlets, the last, as outlet, extracting the foul air, as in the above figure. Through a small grate from three to five cubic feet of air per second will pass, while through the ordinary grate, from five to eight cubic feet; that is to say, in the former from 10,800 to 18,000 cubic feet per hour, and in the latter, from 18,000 to 28,800 cubic feet in the same time. To meet this, air rushes in from crevices around windows and doors and through flooring-joints. The atmosphere of a room is practically divisible into two different layers, the lower reaching from the floor to the level of the artificial lights, and the upper from the lights to the ceiling. By reason of the door and window currents the coldest parts of a room are in lines drawn, between these points and the fire-place, in straight paths. The lower stratum is, therefore, some degrees colder than the upper, and is often too well-ventilated—draughty—while the upper is practically unventilated unless by the diffusion of gases. In a room without a fire the body-heat of the occupants determines the same currents, but at a less rate of motion, and upon occasion, in summer, in basement flats, the chimney may act as an inlet of air. The general principle always to be kept in mind is, that a colder body of air always moves in the direction of a warmer, and with a degree of velocity corresponding to the relative difference of temperature; the greater the difference in temperature the higher the velocity. Ventilation of the sick-room is one of the most important duties of the nurse, and she ought to be able to exercise some ingenuity to effect this object. The first point to consider is the relative position of the bed of the patient to the window, door, and fire-place. If the plan of the room permit, the bed ought not to be placed in a direct line between door, or window, and fire-place. It ought to be in the leeway of both, outside of direct air-currents. If this, however, cannot be avoided, direct draughts must be prevented by the interposition of a screen or curtain. The ventilation of the room may be effected either fitfully, *i.e.*, for brief periods every hour, or steadily and continuously throughout the day and night. Under the former plan probably the simplest and safest way is to half open the door, pull down the upper window-sash for a distance the extent of which must be regulated by outside weather conditions, and cover this by the blind, a few inches below the level of the opened sash, in order to break any direct draught. Protect the bed by a screen. Leave the sash open for about five minutes at a time, and repeat from two to four times per hour. Any plan, however, which would enable ventilation to go on continuously is obviously better. To this end various expedients have been suggested, which are usable in ordinary sash windows.

Fig. 7.

Fig. 8.

A is block of wood at bottom of window; C is the upper part of lower sash; the long arrow shows the direction of air-current, and the small arrow passes through a conical hole in lower part of upper sash—an addition sometimes made, but not suitable for town houses where air contains "blacks." By varying the size of block A, the size of interspace is regulated.

Fig. 8 shows another method of producing a like result. B is a metal plate affixed to upper part of window-frame, which enables upper sash to be pulled down without top draught, and creates interspaces as in Fig. 7.

The plan of Hinckes-Bird is very simple and easily applied. It consists in placing a block of wood, varying in thickness, on which the lower sash will rest, at the bottom of the window. This has the effect of separating the sashes at their junction, and enables air to pass inwards between the sashes. Fig. 7 illustrates the effect produced.

Very little expense and trouble might upon emergency simplify window ventilation. If, for example, Moore's louvre, or circular, glass ventilator were fitted into a window frame, draughtless ventilation may be effected. (Figs. 9 and 10.) The Louvre ventilator may be opened

Fig. 9.

Fig. 10.

according to necessity, and in like manner the circular, which in Fig. 10 is shown closed.

Another excellent plan is to substitute for the upper half of the window Hatton's Hopper Window Ventilator, which not only prevents direct draught, but at the same time screens the air of particles—a desideratum in smoky cities.

Various other modes have been suggested, such as double windows, perforated panes, and double-glazed panes, where the outer pane has a bottom gap and the inner an upper gap; but in smoky places they are decidedly objectionable, because of the "smuts" which accumulate in the interspaces, and which cannot easily be cleaned.

### Trained Nurses' Clinic.

### IV.—THE NURSING OF BRONCHITIS.

Diseases of the bronchi are attended by symptoms more or less familiar to all English people, and few winters, therefore, pass over without a variety of cases of acute bronchitis requiring special attention from nurses, and in addition the usual proportion of sufferers from a chronic form of the disease demand much attention and care. Every medical ward in a general hospital provides abundant opportunities for observing bronchitis in all stages, and in workhouse infirmaries the nurse is soon familiarised with a very severe type of the complaint.

Not only are damp and cold predisposing causes, but also fatigue, the bad air of over-heated rooms, sudden changes of temperature, &c. With adults overwork, or improper food and certain kinds of occupations, are also factors in the causation of diseases of the bronchi.

All such suggestions of predisposing causes demand special attention from nurses, for they belong to that preventable aspect of disease which is too often overlooked by otherwise intelligent persons. Many fail to see that it is wiser to escape by means of care and common-sense the onset of an attack of illness, rather than to drift blindly into danger through neglect of precautions entailing some little personal trouble.

The elementary laws of hygiene are as little understood by the general public as the precise position of the various organs in the human body, and it is no uncommon thing to find a person resent the insinuation that there is nothing whatever the matter with the respiratory organs, but that the digestive tract is alone responsible for certain troublesome symptoms.

Whether on account of their higher situation or of their obvious importance it is difficult to say, but certainly most persons cling to the theory that there is something wrong with their chests even when the doctor has confidently proved the lungs to be sound and the liver or stomach alone temporarily deranged.

Hence bronchitis is not only common, but to a certain extent it is a popular affection, and a district nurse rarely accomplishes her daily rounds without being informed by some anxious mother that her husband or child "'ave got a touch of the *brownchitis.*" The cheerful rejoinder, "I hope not," is invariably passed over, and she may often deem it wise to advise a quiet day in bed, and light food only, until the doctor has been summoned to decide whether the threatened *brownchitis* is actual or imaginary. In any case, for the hard-working and ill-nourished person a quiet day in bed never comes amiss, and is more easily secured than the prolonged confinement to one room which a serious attack of bronchitis may eventually necessitate. Even in the houses of the well-to-do it is sometimes difficult for the nurse to maintain the equable temperature which the doctor desires. The rooms of the poor are crowded with undesirable accumulations which are hard to get rid of, because there is nowhere else to put them. An improvised screen is generally one of the district nurse's first cares, but in her absence this is often removed by the friends who see no harm in letting all the evil odours of the house drift straight in to the patient, whilst they feel confident that nurse's attempts to purify the room by the cautious admission of fresh air are risky in the extreme.

Of course the bed-rooms of the prosperous classes are not overcrowded to the same extent as those of their poorer neighbours, but they are often much too full of superfluous articles, especially in cities where architects are apt to consider lofty and light rooms essential for day use, but low ceilings, small windows, and a dull outlook quite adequate for the majority of sleeping rooms.

If the patient be the head of the household, his own room in its normal condition may be a fairly satisfactory one in which to nurse him; in the case of less important members of the household, and especially servants, the apartment often leaves much to be desired. By the exercise of tact and commonsense a good nurse can generally amend matters, especially if she confines her suggestions to practicable improvements, avoiding the obvious folly of finding fault with irremediable evils.

(*To be continued.*)

### How Small-pox is Nursed in Gloucester.

From a Correspondent.

Anyone who has more than a superficial acquaintance with the ancient cathedral city of Gloucester and its ways must realize that for general provincial ignorance and stupid resistance to modern ideas it has probably no equal in the three kingdoms. Many people have, therefore, not been surprised at the continuance of the present epidemic of small-pox, with little abatement, for over four months; but even to them the condition of things existing in Gloucester is, from a sanitary point of view, almost incredible.

It is true that, at the request of the Guardians, Dr. Sweeting, of the medical department of the Local Government Board, is now vigorously carrying on investigations, and that the superintendence of the temporary hospital has been undertaken by Dr. Brooke, of the Thames Ambulance Service, who has accomplished the appointment of a trained and experienced matron, and secured the services of a certain number of trained nurses; but this advance has been only made quite recently, and instead of assisting to bring things into order the local authorities are annoyed that pre-existing arrangements should have been condemned. The "temporary small-pox hospital" is the ordinary town isolation hospital, situated close to houses and in the town. To this original building block after block has been added to meet present emergencies, till every available bit of ground is covered. A caretaker and his wife were for weeks in sole charge, and all the cooking was done in the wards by the nurses, of whom only a very few had any real claim to the title. The laundry has been crowded with the washing of weeks; bath-rooms were filled with bundles of soiled linen and the clothes of the patients who had come in, and rubbish of every kind was flung outside the hospital. There was no porter, anyone with or without character was taken on to do the "nursing," sharing the same rooms with the servants, and even the same beds. There has been no night superintendent, a discharged patient acting as male attendant at night, and in some wards of thirty odd patients there was no trained nurse at all. It is small wonder that the hospital has been in the worst possible odour among the poor people (who have positively refused to enter its doors), for it cannot be denied that many patients must have died for sheer want of proper attention. The wards have been much overcrowded, often two children in a bed.

There is another temporary hospital at Hempstead, one and a half miles from the city, at which some of the Clewer sisters have been working bravely against almost overpowering difficulties. The hospital is built in a rough field, with no drainage, and no water supply except that brought by carts daily. The overcrowding here, too, has been terrible, and now no further cases are being taken in at the hospital. The lack of proper nursing has not been because volunteers were few. Nurses are ever ready to come forward in times of epidemics, and the present has been no exception to the rule.

It is well known in Gloucester that the opinion is held by some in the city that small-pox needs no nursing, that anyone can attend to the patients, and also that they should not be washed till convalescent; so the condition of the unfortunate victims to this most horrible disease, upon whom this enlightened treatment has been tried, may be better imagined than described; and, high as the death-rate has been, it can only be wondered that it has not been higher.

It is a comfort, in the midst of a condition which is a scandal and a disgrace to a civilised town at the end of the nineteenth century, to turn to the organisation of the district nursing, which has been admirable. The lady superintendent has a special staff of twenty-one nurses working under her, the committee is an excellent and enlightened one, and funds have come in well, fortunately, for it is to be feared that the work of getting the upperhand of the epidemic is by no means at an end; more cases have been notified this last week than in the one preceding. There is a grim poetical justice in this ghastly celebration of the centenary of the discovery of vaccination, but if ever a town deserved retribution, that town must be acknowledged to be Gloucester. It has been doing its best for years past to bring about the fate which has now befallen it.

### Sir Benjamin Richardson on Nurses' Pensions.

In the May number of *Longman's Magazine* Sir Benjamin W. Richardson discourses in a kindly and sympathetic manner on the sick nurse, her duties, her dangers, and what is to become of her when too old to work. It certainly cannot be said that his article shows any wealth of knowledge of the inner life of the nursing world, and it is somewhat startling to find the author of an essay which leads up logically to its inevitable conclusion that a sick nurses' pension fund is an absolute necessity should have to confess in a footnote that he did not know of the existence of the Royal National Pension Fund for Nurses. Nevertheless, as showing what the public ask for in their nurses, the article is of much interest, for truly they seem to demand the very cream of a woman's life—a demand which may well justify nurses in asking a remuneration greater than a mere pay for the number of hours spent, and a consideration in the way of pension far greater than is accorded to workers in fields in which it may be possible to find remunerative employment perhaps during a period of forty years. Sir Benjamin is undoubtedly expressing the common view of those by whom the modern nurse is employed when he says that she should not much exceed forty years of age; that she should be good-looking and active; that all her senses should be perfectly acute; that she should see well, hear well, and touch well; that she should be ready at all times for all services that may be demanded of her; that she should be skilful and experienced; that she should not only be an accurate observer, but able to report intelligently to the doctor; that she should be educated in everything that relates to disinfection; and that she herself should be of neat and comely aspect, cleanly, well dressed, and of cheerful mind and character. But to ask for all this is to ask for the very best of womanhood, and, having got it, to take out in the nursing service the very best years of her life—viz., from twenty-five to forty—and then to turn her adrift. Nothing could express more forcibly than these definitions of what a nurse should be the absolute cruelty of the system at present in force, according to which, to meet the demands of an exacting public, young women are drawn into nursing work, tempted at twenty-five years of age to enter on a career the mere training for which occupies three years, and then, even by kindly sympathisers, voted old at forty, and within a few years afterwards turned adrift. But, on the other hand, nothing could better put before the nurses who are now growing up the absolute necessity of their joining the Royal National Pension Fund while they are young and healthy. In hospitals, perhaps, nurses may not often be openly dismissed on account of age, although they are cruelly apt to slip out of the ranks and find themselves superseded when knees become stiff and backs get weary. In private work, however, there is no reticence or hesitation; the demand is for the strong, healthy, and "comely" nurse, and women who have given their best years to the work, and have too often frittered away their earnings in charity and well-earned holidays, find themselves at forty-five pushed aside by their younger sisters, and, unless they have joined the pension fund, left to penury in old age.

### The Summer Exhibitions.

The New Gallery.

Judged as a whole, the exhibition at the New Gallery is above the average, for whilst it cannot, perhaps, boast of any one supreme piece of work, it is distinguished by a good all-round character. There are scarcely any really bad pictures here, and we notice that one marked feature in this year's art is a general effort towards a higher standard of subject picture than was the case in the exhibition of twelve months ago. It would also point to a brighter state of the picture market; for the New Gallery is rife with work which is of the venture kind, as opposed to the "order." Of course there are portraits, but they do not form the principal part of the show; there are some excellent examples, but subject pictures and landscapes are better represented.

Of the portraits, Mr. Sargent's "Countess Clary Aldringen" (240) is the most striking. Countess Aldringen is represented life size in a white sheeny satin gown, standing facing us; she has evidently just risen from the sofa behind; she is smiling, and her face suggests that she is receiving some approaching guest. The canvas is impressed with the stamp of the painter's inimitable mastery over the technicalities of his art. Mr. Shannon has two good portraits, but we have seen better results from his brush. Alma Tadema's "Family Group" (No. 87) is a careful piece of work, on some unattractive faces of various ages, remarkable principally for similarity of feature and colouring; but the picture has its points.

Of the subject-pictures, those by Burne Jones command one's first attention. There are only two this year, and both are in the West Room, the one, "Aurora," occupying a central position on the west wall, the other, and the larger, "The Dream of Launcelot at the Chapel of San Grael," the central position on the north wall. The former, "Aurora," though a sad representation of the goddess, usually shown in glad joyance, is an imposing picture. Aurora is sounding her awakening cymbals, in the early, fresh morning; she has not awakened her world yet out of its quiet sombre stillness. The sky is illumined, nothing else. The coursing stream by which the goddess walks is gray, so are the mediæval houses; it is an allegory of Dawn in its yet early stage, and in conception is weird, mysterious, suggestive. "The Dream of Launcelot," less imaginative, perhaps, is a perfect specimen of the great master hand. The man's attitude betokens deep-felt shame; so also does the woman's; and the picture is intentionally sombre.

The Hon. John Collier's "4 a.m." (No. 255) is in the North Room. A clever, bright, attractive study this is of two girls talking over a dance, which is evidently just over. The one, in her ball-room attire, programme in hand—the other is in bed, listening. The whole is vividly life-like. Last but not least among the great subject pictures (many more of which deserve full mention) are Watts's "Time, Death, and Judgment" (No. 79) and "The Earth" (No. 67). Whether the latter finds favour or not is a matter of individual opinion. It is a study which grows on one the more one looks, and is brimful of noble, inspiring suggestiveness. The artist said of his works, "I paint ideas, not objects"; "I lead men to the Church door, and then they can go in and see God in their own way."

Landscape is unusually strong in the New Gallery exhibition this summer, but though space forbids individual mention of works which are, in many instances, quite above the average. One cannot fail to observe a growing feeling after the old schools; and this gives to several conspicuous landscape studies in the New Gallery a strained, unnatural effect. One or two of these larger canvases, lacking all direct spontaneousness on the part of the painter, give the impression of discipleship, and nothing else. Moffat P. Lindner's "Autumn" (No. 212) in the North Room stands out in striking contrast to its surroundings, through its strong, bold independence of method. It is the most entirely original picture in the gallery, if not in its conception, certainly in its execution.

### National Hospital for the Paralysed and Epileptic, Queen Square.

A pleasant concert, provided by the Countess of Bantry and Miss Allanson-Winn, assisted by Madame Mera and Captain McLaughlin, R.H.A., took place on the 30th ult. Afterwards the prizes won at the annual examination by the probationers training in the hospital were presented by Lady Bantry. Mr. Burford Rawlings, in a few introductory words, observed that the hospital had the unique distinction of training men as well as women nurses, and he thought it would be regarded as a somewhat remarkable fact that the chief honours in the present case had been carried off by the men. No one would deduce from this that there was any danger of men supplanting women in the field of nursing, but that there is room for a certain number of male nurses, and that it is a pity the general hospitals do not help to train them are facts very generally admitted. The National Hospital, thanks in a great measure to the co-operation of trained women, had not only sent out a considerable number of well-trained men, but it possessed a small staff of male nurses working in the wards whose aptitude and efficiency left little to be desired. Mr. Rawlings concluded by a tribute to nurses in general, remarking he thought it well to hold them in honour, to offer them never-failing sympathy and encouragement, and even admiration, because a really good nurse approaches very nearly to a good and perfect woman. Lady Bantry then presented the prizes, consisting of suitable books, to the successful competitors, who were severally introduced by the Lady Superintendent, to each addressing a few kind words. The awards made by Dr. Whiting, the examiner, were as follow: Senior physiology, Probationer Male Nurse Kingaby; junior ditto, Probationer Nurse Goy; senior electricity, Probationer Male Nurse Kingaby; junior ditto, Probationer Nurse Museen.

### Where to Go.

St. Martin's own Hall.—The Duchess of Teck presides over the opening of a bazaar on May 18th in aid of the Royal Eye Hospital, Southwark. The opening is at half-past two, and the bazaar continues on the following two days.

Westminster Town Hall.—A concert is to be given by the Children's Orchestra in aid of the Farningham Homes for Little Boys on Monday, the 18th, at eight p.m. Mons. Johannes Wolff and Mons. Joseph Hollman have promised their services. Tickets, 7s. 6d., 5s., and 2s., may be obtained from the Secretaries, Homes for Little Boys, 25, Holborn Viaduct, E.C.

### Minor Appointments.

Denbighshire Infirmary.—Miss Enid Ellis has been appointed Head Nurse at this infirmary. She was trained at the Stanley Hospital, Liverpool, and has since worked as charge nurse at the City Hospital, East Liverpool.

Chorley Dispensary and Cottage Hospital.—Miss Louisa Dawson has been appointed Sister-in-Charge at this dispensary. She was trained at the Royal Albert Edward Infirmary, Wigan, afterwards working in the same institution as staff nurse, and as night sister.

Macclesfield General Infirmary.—Miss Ruth Summers has been elected to fill the post of Night Superintendent at this hospital. She received three years' training at the Manchester Royal Infirmary, afterwards acting as sister at Professor Sinclair's Private Hospital for Women, and as sister of the female wards and theatre at the Macclesfield General Infirmary. We congratulate Miss Summers on her promotion.

### A Book and its Story.

"THE YOUTH OF PARNASSUS."*

The scene of Mr. L. Pearsall Smith's tale is laid at Oxford, something of the spirit of the old University town permeates each page, and a sense of reality stamps each of his descriptions of the undescribable city. For Oxford is undescribable, Oxford life, anyhow. If, in one phase, it has been shown up in literature, in all its versatility it never has. That Mr. Pearsall Smith has been *in* Oxford, "of" her, admits of no doubt whatsoever. In the "Youth of Parnassus" he brings before his readers much of the quiet and peace of the old world city, with its inexhaustible wealth of interest, human and architectural. In a subtle manner, too, he shows the influence that Oxford has upon its sons, a power which operates in many varied ways. In the "Youth of Parnassus" he describes how the traditions of the past worked on a mind fresh from a modern Republican country.

This is the history of a serious-minded young American man, who, at the instigation of an undenominational order in his native country, is sent to the great English University. The book opens with his arrival at Oxford from Parnassus City, a town in the Western State of Indiana. He is described as a young man who wore spectacles and had a pale face. To Foley (a fellow-undergraduate who lives, at college, in the rooms beneath him), who came to call, he seemed nothing but the traditional Western American he had read of in books or seen in the theatre sometimes—a student who looked curiously out of place in that panelled room. The young Englishman talked to him during this visit as best he could, asking the questions always asked of a new comer, questions answered by Sutton in a shy and diffident manner. "He had come to study under Dr. Joseph," he explained, "at the New Methodist College. Dr. Joseph had arranged for him to come to St. Mary's; their own college wasn't built yet." Foley asked if he thought he would like Oxford. "Yes, sir," the other replied, drawing a large handkerchief from his coat-tails, "I guess I will; though," he added cautiously after a moment, "it does seem kind of old and mouldy."

Foley thought he had done his duty in calling, and meant for the future to see as little as possible of his new neighbour. And yet there had been something pleasant and sensitive in his face he remembered afterwards, and at times he was "haunted by the thought of the stranger, sitting as he had found him, alone and lonely in the room upstairs." This interest in the new comer grew, and before long Foley is shown as taking a certain liking to Sutton. The young American presented a curious and amusing study to the English undergraduate; he would try and draw out his opinions of Oxford. The youth admitted that, "Of course, there was a great deal of culture in Oxford; but in religion, as in all other things, like telephones and electric lights, why England was behind the Mississippi valley."

One evening, in a burst of unusual talkativeness, Sutton tells his new friend a little about this wonderful Parnassus City; how it had been laid out twenty years before, on what had been an unploughed prairie; but now there were "thousands of inhabitants, rows of business buildings, and elegant residences in the outskirts, electric trolleys, &c."

And in the culture of the place, the growth had been equally rapid, its champion declared. Schools and churches were springing up, the First Methodist Chapel, the Reverend Dr. Turnpenny's, being the most elegant. This Dr. Turnpenny it was who had started the Forward Movement among the Indian Methodists. A photo of Parnassus College was pinned against the wall. Foley looked at it. A gaunt, new building standing in a ploughed field, a few young trees around it. On the steps of the college were grouped a few human beings. "That's my class," Sutton explained, "it's the biggest class we've had so far, thirteen gentleman and seven ladies." And from this talk of the wondrous Parnassus, Foley turned; going to the window he opened the lattice and leaned out into the night. "Cool, fresh, and dark was the air that breathed on his face, while before him, blue and vague under the white moon, there grew on his sight the towers, the dome-like trees, and the shining roofs of Oxford—dim, romantic, and steeped in silence, save for the even tinkle of a distant bell. With sudden unaffected sentiment he felt how much he cared for Oxford, and all that Oxford stood for."

"Do come here," he called out with a friendly impulse, turning his head into the yellow light of the room; "I don't think I ever saw such a view." "Yes, it is nice," the American said at length, and Foley was surprised at a fugitive sound of real feeling and appreciation in his voice.

If only this young man could be made to look at life for himself thought Foley, instead of seeing it through the grey fog of Puritan prejudice! Foley had Ritualistic tastes; Sutton would give him tracts to read. This was at first. Little by little the American modified his views as time went on; but the Puritan prejudice against enjoyment of life still dominated his being; he would remain indoors absenting himself from all possible pleasure.

Then Foley took it upon himself to force Sutton to see more of the world and life. "Living in his lonely, retired way, what could he know of other people and the things they cared for, and how could he ever hope to have any influence upon them?" he said. Perhaps, he sometimes fancied, Sutton had no real ideas and impressions of his own; perhaps he was not influenced by Oxford in the least, and was not aware of any real difference between the ancient town, with its traditions and memories, and the new-built Parnassus City. And yet a change was coming over the young man—he had come to see "the propriety of being familiar with modern views and modern books." He went so far as to see also "the advisability of trying to understand one's opponent's views." Letters at this time came from Oxford to Foley who was abroad; letters of a touching and intimate nature. Sutton was "regularly attending the college service now," he wrote. Sutton was staying on at college, having friends nowhere else. Oxford was widening the young American's views. He even surprised himself by admitting that there wasn't any danger in the new criticism—there never could be any real danger either, he said, "in following one's best reasons, and we need not be the least afraid of what it will lead to."

And through these letters there ran a thread of almost enthusiastic love for the city of his adoption; her spell was full upon the young man's senses, the sights and sounds of Oxford had gained an entire hold upon his being; a passion for the place breathed in Sutton's letters, his growing preoccupation with, and interest in, everything that was ecclesiastical and ancient.

The change in the young man was gradual, as it was sure. It is described with a masterly hand, and is the *raison d'être* of the book. The story presents a metaphysical study of the workings of the young Dissenter's mind; his struggles to be true to his master; his struggles against the growing hold the past has worked upon him. In the end these latter conquer. The Puritanical mind of Dr. Turnpenny's protégé is changed; he is a Roman Catholic. Foley expostulates with Sutton; he should go home, he urges, and let work and change of scene act upon his temporary want of allegiance to the religion of his youth; some compromise might be entered into. "No," cried Sutton, "we can make no compromises; we must give up human reason; we must go back to the past; we must submit." Then Foley appeals to his better sense of right—Dr. Turnpenny's claim upon him. "I could not go back to them a Roman Catholic," the other answered. "They would rather I was dead. Remember, Foley, when you judge me, that I have had my sacrifices, too. I have given up everything—everything; and you don't understand," he said in a voice that his friend remembered afterwards, and then he went down the path alone, and out of Foley's sight.

* "The Youth of Parnassus." L. Pearsall Smith. (London: Macmillan and Co., 1896.)

### Novelties for Nurses.

AT MESSRS. DEBENHAM AND FREEBODY'S.

Messrs. Debenham and Freebody have lately opened a new department in Wigmore Street which is entirely given over to the various requirements of the nursing profession. Nurses' uniforms, cloaks, shoes, caps, aprons, wallets, instruments, &c., are here displayed in a handsome apartment, fitted up with comfortable chairs and sofas, and well provided with the current periodicals. Their new department is of so attractive a nature one cannot fail to comment upon it; and we must congratulate the firm on the successful issue of their endeavour, for their thoughtful arrangements are calculated to make shopping in Wigmore Street something other than a disagreeable duty. And now, to descend to particulars of what we specially noticed among the nurses' requirements, we will commence first with the cloaks. The selection of these has been carefully designed and made with the idea of providing a thoroughly practical garment suitable for town or country wear. These are made up one and all in good and servicable shower-proof materials, well cut, and neatly finished off. Among these the "Sister Dora" and the "Cavendish" have proved most popular, for whilst keeping strictly to the requirements of hospital uniform, they give free play to the arms, and are quickly put on. These are both made without capes, but there were plenty of other designs with the cape, cut to admit of the fullest of undersleeves. The uniforms, which are noticed as ready kept in stock, were in serge, and others in fresh pretty zephyrs and ginghams. Messrs. Debenham and Freebody make a special point of the quality of their dress materials, all of which are tested before making up. The firm are designers of nurses' uniforms, and are prepared to submit original designs for hospitals and nursing institutions requiring distinctive uniform. Messrs. Debenham and Freebody were selected to make the Queen's Jubilee nurses' uniform, which has received the approval of Her Majesty. So, as we said, though a large selection of dresses are kept in stock, yet their customers are not limited to these on show. A register of all the various hospital uniforms, carefully indexed, is kept in Wigmore Street, which facilitates orders by post receiving prompt attention. Several novelties in caps and aprons attracted our attention, some of the former being prettily embroidered in lawn or muslin. Nurses' bonnets are stocked in numerous shapes, the "Sister Dora," to be worn *en suite* with the cloak of the same name, being a fine black straw, full velvet lining over the brim, velvet bow and string, and white cap front.

### Nursing in Buenos Ayres.

A correspondent who has lived for some time in Argentina sends the following information with regard to the prospects of private nursing in Buenos Ayres, as to which a query appeared in the "Nursing Supplement" a few weeks since. A nurse holding a certificate for midwifery wishing to practise in Buenos Ayres should see the Argentine Minister, as he would be required to countersign the certificate on the same lines as doctors' diplomas. She will also be required to go through a short examination in Spanish or French, attend a few cases, and pay a fee of fifty dollars. I have been told after passing the examination nurses are not allowed to refuse any case that may come to them. Within the last few years two English midwifes have been working in Buenos Ayres. Dr. Cecilia Grierson is very kind, and would give any help or information. For some years she has had native women training as nurses in the native hospitals. With regard to obtaining quarters, it is certainly unwise to venture into foreign parts without an idea of where to go. Associates of the Girls' Friendly Society would help in this way and with advice. Nurses need money in hand, as they would have to wait their turn on the doctor's books, and could only hope to attend people speaking English. Living, too, costs twice as much as in England.

### Reading to the Sick.

ASCENSION-TIDE.

**Motto.**

In contemplation of created things
By steps we may ascend to God. —*Milton.*

**Verses.**

Nor doubt that golden corde
Of good worke, mingling with the visions, raise
The soul to purer worlds. —*Wordsworth.*

God often would enrich, but finds not where to place,
His treasure,—nor in hand nor heart a vacant space.
—*Hymns Ancient and Modern.*

See, the Conqueror mounts in triumph,
See, the King in royal state
Riding on the clouds His chariot
To His heavenly palace gate.
Hark! the choir of angel voices
Joyful alleluias sing,
And the portals high are lifted
To receive their Heavenly King.

He has raised our human nature
On the clouds to God's right hand;
There we sit in heavenly places,
There with Him in glory stand.
Jesus reigns, adored by angels;
Man with God is on the Throne.
Mighty Lord, in Thine Ascension
We by faith behold our own.

O Saviour, who for man hast trod
The winepress of the wrath of God,
Ascend, and claim again on high
Thy glory left for us to die.
O Christ, our Lord, of Thy dear care
Thy lowly members heaven-ward bear;
Be ours with Thee to suffer pain,
With Thee for evermore to reign.
—*Hymns Ancient and Modern.*

**Reading.**

"I ascend unto My Father and your Father, to My God and your God."—*John* xx. 17.

"Who shall ascend unto the hill of the Lord, or who shall stand in His Holy place? He that hath clean hands and a pure heart."—*Psalm* xxiv. 3, 4.

What the first Whitsunday was to all the world, one certain day becomes to any man, the day when the Holy Spirit comes to him. God enters unto him, and he sees all things with God's vision. Truths which were dead spring into life, and are as real to him as they are to God. He is filled with the Spirit, and straightway he believes; not as he used to, coldly holding the outsides of things. He has looked right into their hearts. His belief in Jesus is all afire with love. His belief in immortality is eager with anticipation. Can any day in all his life compare with that day? If it were to break forth into flames of fire, and tremble with sudden and mysterious wind, would it seem strange to him—the day when he first knew how near God was, and how true truth was, and how deep Christ was?

Let us pause a moment and think what Whit Sunday was, the first Whit Sunday. We read the story of the miracle. We hear the rushing of the mighty wind and see the cloven tongues of fire quivering above the heads of the Apostles. Perhaps we cannot understand it. . . . It was the coming back of God into man. It was the promise of these typical men of how near God would be to every man henceforth. It was the manifestation of the God Inspirer as distinct from and yet one with the God Creator and the God Redeemer. It was primarily the entrance of God into man, and so, in consequence, the entrance of its spirit and full meaning into every truth that man could know. It was the blossom-day of humanity, full of the promise of unmeasured fruit.—*Phillips Brooks.*

### Everybody's Opinion.

[Correspondence on all subjects is invited, but we cannot in any way be responsible for the opinions expressed by our correspondents. No communications can be entertained if the name and address of the correspondent is not given, or unless one side of the paper only be written on.]

### DIFFICULTIES OF PROVINCIAL MATRONS.

"A Staff Nurse" writes: A correspondent writing in The Hospital of April 11th, under the heading of "Difficulties of a Provincial Matron," must have had an unusually unfortunate experience. In the first place, it is well known that in provincial hospitals, at least in those of any size or standing, the nurses are more select, as a rule, than in most of the large town hospitals. This matron does not give one the impression of having had much sympathy with her new charge. Some matrons seem entirely to forget that they have been nurses themselves, and treat those under them as inferior beings. Surely, in the "fine London ward," where your correspondent was "sister," the matron did not pay her a visit when the doctors were going their rounds—at any rate it is not usual in Scotch hospitals. If anyone wishes to be both nurse and matron they should take a cottage hospital. As to the doctors, they cannot be expected to have much respect for one who treats them with "contempt." With a little tact and forbearance one can get on with most people.

### YOUTHFUL NURSES.

"H. C." writes: Will you allow me through your columns to give my experience in private nursing to young would-be probationers who wish to begin training before they are twenty-one? Like many a young girl, I was most anxious to get into a hospital as a probationer. I applied to hospitals far and wide unsuccessfully. I had gained a "First Aid" certificate, and had good testimonials both from a doctor and a clergyman, but still, as I was only nineteen, I had to give up all thought of nursing for a year or two. Then, greatly to my joy, a lady asked me to attend her sister (a slight mental case). Glad to do anything in the way of nursing I went, and my work for a long time was very easy. But latterly my patient became very hard to manage, gave me many a hard blow, and played many a trick on her poor maid. Three times I gave up my work, but in the end was persuaded to stay, the doctor always saying it would not be for long (the lady was then seventy-five years old). However, she still lives, while I have been in bed for ten weeks owing to the treatment I received from her, and never, the doctor tells me, will I be strong enough to undertake the duties of a sick nurse. So I would advise all would-be probationers never to begin nursing until they have reached the age of twenty-one. Of course, things are different in a hospital, but still I think girls should never go in for training till they are at least twenty-one or twenty-two. All the thanks I got from my employers were, "I am sorry your health has given way, but you were far too young." I knew it was foolish of me to undertake a mental case at nineteen, but one is glad to find work one likes, especially when something must be done to earn a living.

### THE NURSE GOSSIP.

"Justitia" writes: It has been to me a matter of much surprise that none of your nurse correspondents have taken up the cudgels on behalf of their profession, in view of the above accusation. Who is responsible for the creation of the nurse gossip? Why, the patient of course, who, in nine cases out of ten, develops during convalescence an unnatural appetite for morbid horrors and sensational tit-bits of well-spiced gossip. Particularly so, when the latter are concerned with that portion of society which is known as smart. Should the nurse fail to provide this form of entertainment to Mrs. Jones of Clapham, or Mr. Brown of Stoke Newington, they feel themselves aggrieved and pronounce the nurse as being "no class," it being in their eyes an acquired distinction to have been nursed by the same woman who attended Lady A. or Captain B, "of whom my nurse has told me such dreadful things, you know," and then follows a horrified shudder over "wickedness in high places." But even Philistine strongholds have their family skeletons, which the nurse may have seen in all its vertebrate entirety. Whereupon these estimable people suddenly bethink themselves that its unmasking may yet furnish sport for other Philistines. So they unblushingly brand the nurse as an unprincipled gossip. As a veteran nurse of nearly fifteen years' standing, the greater part of which has been passed in the sick-room of the private patient, I have always found that patients and their friends seem to consider that medical topics only are likely to prove acceptable to the nurse; consequently all the ailments past and present of the family constitution are trotted out one by one, discussed in all their bearings physiological and pathological, what the doctors, surgeons, and nurses said concerning each. Then the nurse is asked to give her opinion, a position which is, to say the least, embarrassing, and from which I extricate myself by a liberal use of medical dictionary phrases which impresses my hearers without committing myself to anything in particular. I am strongly of the opinion that a nurse's moral debasement begins from the time she takes up private nursing; then for the first time she hears delicate subjects discussed with a freedom and frequency unknown in ward life, the chief offender in that respect being the aggressively virtuous British matron. My advice to my colleagues is that whilst in the sick-room a sphinx-like attitude should be adopted, giving the impression that if they chose to reveal the secrets of their profession "they could a tale unfold." But from my knowledge of sick rooms and their occupants, notwithstanding all this outcry about nursing gossip, if nurses took the above attitude there would soon be a "slump" in nursing circles. For, whilst confined to the four walls of the sick-room, the patient directly encourages gossip and expects to get it. It is only with the return of rude health that the "nurse gossip" is repudiated.

### ASYLUM NURSING.

"An Old Asylum Nurse" writes: A few months ago I was very much interested in reading some letters in The Hospital about asylum nurses. I should have liked then to have had my say, but it would have been against the rules of the asylum in which I was working at the time. Now, however I have left asylum life, I will, if you kindly so permit, endeavour to put before you as briefly as possible the life of an asylum nurse. Of course I am only speaking of county asylums. We have heard all about the bad food, the knocking about by patients, and so on. What I want to draw your attention to is the amount of work one nurse has to do, and what she has to put up with from the petty officers. Of the matron I can say nothing; I believe she has much to contend with, and really more than she can do. Ten patients are supposed to be allotted to one nurse, but this is an elastic limit. There are more often fifty patients to four nurses in one ward, that ward being formed of a day-room, sometimes a gallery attached to it, dormitory, lavatory, and bath-room. All this must be done at a certain time. It is said that patients do some of the work; sometimes they do, but then sometimes they don't. At half-past ten two nurses are supposed to go out in the airing court with a certain number of patients. What the officers think chiefly about is the cleanliness of the place, but I have often wondered if they could have told how the work was to be done. Let me tell you one thing, that as long as an asylum nurse has dressmaking to do for the patients, and is made responsible for the house work, it is impossible for her to be as kind as she ought to be. I could name if I liked five different county asylums where different punishments are used by the nurses for the patients. I ask what are we to do when we know the work must be done, and if patients are "playing up," as we call it in asylums, and very little help given from the medical officers? As long as superintendents are not more particular about whom they engage, as long as nurses are treated as they are by the officers, and worked incessantly for fourteen hours a day on bad food and bad pay, nothing can be done. Not only do the nurses suffer, but the patients do too, more than people may think. It is of no use making the rules harder and harder every day, as is sometimes done. Let asylum nurses be only nurses, and not general servants and dressmakers; provide wardmaids and needlewomen in the work-room, and I would venture to suggest Bible classes for the staff (which many ladies would gladly undertake), and then see if better-class nurses will still look down upon asylum work! Indeed, there is work there to be done, work which none can imagine until they enter upon it.

### The Book World for Women and Nurses.

[We invite Correspondence, Criticism, Enquiries, and Notes on Books likely to interest Women and Nurses. Address, Editor, THE HOSPITAL (Nurses' Book World), 428, Strand, W.C.]

MINOR PUBLICATIONS.

"DEVOTIONAL AIDS" has now passed into a second edition, and we are not surprised to hear this little book has met with appreciation from the public. It is a handy little volume, published by James Parker and Co., of Oxford and London, and is offered at the modest sum of 1s. net. It was designed to provide short daily readings, meditations, prayers, &c, for sufferers in times of weakness; it is also interspersed with blank pages.

Another book also somewhat of this order is before us for review entitled STRAY THOUGHTS FOR INVALIDS, by Lucy H. M. Soulsby, and published by Messrs. Longmans and Co. at 2s. The book is very nicely got up, the print being large and the paper good. It is not in the form of a daily manual, but desultory thoughts and verses from varied authors are collected together and agreeably presented to the invalid. Every sick-room would be the better for Miss Soulsby's "Stray Thoughts."

THE COMBE PARK TRAGEDY, by Edith Long Fox. A second edition has just appeared of the above little volume, which is published by J. Baker and Son, of Clifton. The title has its attractions for a certain class of readers, and the entire improbability of the plot has apparently ensured it some success. We use the term "improbability" in lieu of stronger language, and we can only surmise that the authoress of the story is labouring under the disadvantages of want of any literary experience. But, as we have remarked already, the book has passed into a second edition, which is its own hall mark of popularity, and we must congratulate the writer on the successful results attending her effort.

IN PHOTOGRAMS OF 1895 (published at 2s. by Dawbarn and Ward (Limited), of London, we have a beautiful drawing-room album, though it only describes itself as "A Poetical and Literary Record of the best Photographic Work of the Year." In this manner an historic value is given to the production, though the artistic and æsthetic merits of the book will prove enough attraction. Some of the pictures are so beautiful that purchasers of the book have cut them out for framing purposes. They are in all cases reproduced most perfectly, both as regards landscape and figure subjects. This is only the second year of the editor's attempt to produce an annual recording and illustrating, as far as it is feasible, the position of photography up to date. We predict all success to the present venture, which the book most undoubtedly deserves.

### Appointments.

KENSINGTON DISTRICT NURSING ASSOCIATION. — Miss Elizabeth Brook, who has been nurse in this association nine years, and senior nurse for seven years, has been appointed Lady Superintendent, *vice* Miss Ada Booth, resigned.

ROYAL SEA BATHING INFIRMARY, MARGATE.—Miss Heathcote has been appointed Matron at this hospital. She was trained at St. Bartholomew's Hospital, and has since held the appointment of matron at the Hospital of St. Cross, Rugby.

HULL CORPORATION SANATORIUM.—Miss Harriet E. Bland has been appointed Lady Superintendent. She was trained at the Sheffield General Infirmary, and has, besides having had considerable experience in private nursing in connection with the Sheffield and Huddersfield Nursing Institutions, held the posts of sister of male surgical wards at the Sheffield General Infirmary, and matron at the Beverley Dispensary and Hospital.

### Wants and Workers.

A CORRESPONDENT is anxious to know something of the "Delsarte System" mentioned in a little publication called "Power through Repose," by A. P. Call. Can any reader supply information on the subject?

### Presentations.

AT the Burnley Victoria Hospital, on May 5th, Charles Wilson, M.B., C.M. (resident medical officer), was presented by the nursing staff with a handsome travelling clock and brass inkstand as a "token of respect and esteem" on his departure from the hospital, where he was much liked by everyone with whom he worked, and where his loss will be sincerely felt.

AN interesting ceremonial took place at the Macclesfield Infirmary on the 11th inst. The Mayor and Mayoress and Miss Hill met the staff in the matron's room, in order to give them an opportunity of formally taking leave of Miss Bowman, the retiring matron, and offering her their united congratulations on her approaching marriage. Miss Bowman was presented with a black ebony set of toilet requisites on behalf of the sisters and nurses, and with a silver duplex lamp on behalf of the household staff.

### Notes and Queries.

The contents of the Editor's Letter-box have now reached such unwieldy proportions that it has become necessary to establish a hard and fast rule regarding Answers to Correspondents. In future, all questions requiring replies will continue to be answered in this column without any fee. If an answer is required by letter, a fee of half-a-crown must be enclosed with the note containing the enquiry. We are always pleased to help our numerous correspondents to the fullest extent, and we can trust them to sympathise in the overwhelming amount of writing which makes the new rules a necessity. Every communication must be accompanied by the writer's name and address, otherwise it will receive no attention.

**Queries.**

(38) *Disinfection.*—Will you please give me the address of a reliable place in London where nurses can be sent to disinfect after fever cases, if possible, easily accessible from Blackheath? Is there any such place near Richmond?—*Lady Superintendent.*

(39) *New York.*—Please tell me the name and address of a nursing association in New York to which I might apply? I am a certificated monthly nurse and masseuse.—*Nurse H.*

(40) *Medical Training.*—Please tell me how I can become a lady doctor?—*A. A. H.*

(41) *Training.*—Where can I obtain sufficient training for private nursing, in or near London?—*Enquirer.*

(42) *Asylum Attendant.*—Will you kindly tell me what qualifications are necessary for a "certificated lunatic attendant"?—*Incognito.*

(43) *Gynæcological Nursing.*—Can you give me some information about hospitals for the treatment of gynæcological cases only? I have looked in "Hospitals and Charities," and am surprised to see how few such institutions there are. Can you tell me about women's hospitals abroad?—*Nurse Catherine.*

(44) *Monthly Nursing.*—Can you tell me of any co-operation or institution that provides monthly cases for independent private nurses on commission?—*Ettie.*

(45) *Invalid Cookery.*—Would you kindly let me know through your columns of any classes in invalid cookery which a friend of mine could attend in an afternoon?—*Sister Grace.*

**Answers.**

(38) *Disinfection (Lady Superintendent).*—We believe Miss C. J. Wood, Nurses' Hostel, 27, Percy Street, Tottenham Court Road, W.C., takes nurses for disinfecting and quarantine. We do not know any place near Richmond where such facilities are offered.

(39) *New York (Nurse H.).*—There are many nursing associations and directories in New York, but Englishwomen, even thoroughly trained nurses, would stand very little chance of getting private work through them. There is quite as much competition in America as in England, and you would be most ill-advised to attempt emigrating to New York on the chance of finding work.

(40) *Medical Training (A. A. H.).*—Write to the Secretary, London School of Medicine for Women, 30, Handel Street, Brunswick Square, W.C.

(41) *Training (Enquirer).*—What do you mean by "sufficient training?" If you wish to take up nursing at all there is only one proper course, and that is to enter a general hospital for two or three years' training. You will find a list of training schools, London and provincial, in Burdett's "Hospitals and Charities." Write to the matrons for forms of application and regulations. Probationers are paid a small salary.

(42) *Asylum Attendant (Incognito).*—Apply to the Medical Superintendent, Berrywood, Northampton.

(43) *Gynæcological Nursing (Nurse Catherine).*—There are in most general hospitals special wards devoted to such cases, which obviate the need for a multiplication of special hospitals. If you want to work in a special women's hospital, why not apply at the New Hospital for Women, Euston Road? It is not so easy to obtain admission into foreign hospitals; your best way to gain information is to write for particulars direct to the various institutions which you find mentioned in "Hospitals and Charities."

(44) *Monthly Nursing (Ettie).*—Each of the lying-in hospitals keeps a register of the nurses trained by it, and charges a small commission on cases. Members of the Midwives' Institute and Trained Nurses' Club also can enter their names on the register kept at the club. Write to the Secretary, 12, Buckingham Street, Strand, enclosing stamped envelope.

(45) *Invalid Cookery (Sister Grace).*—Write to Miss Halliday, 6, Arlington Street, Piccadilly, who has lately been holding classes for the nurses of the Metropolitan Nursing Institution.

## Figures and Tables

**Fig. 5 f1:**
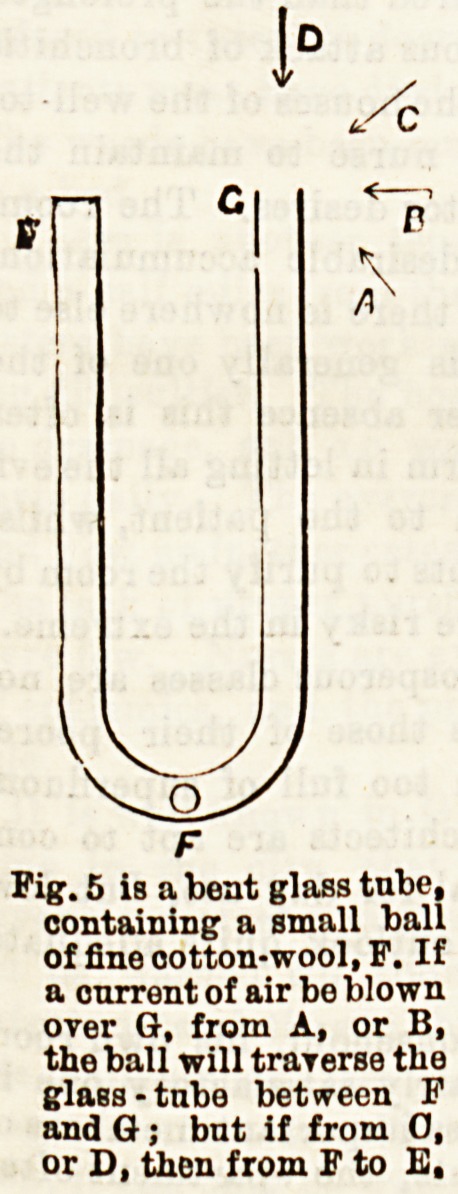


**Fig. 6. f2:**
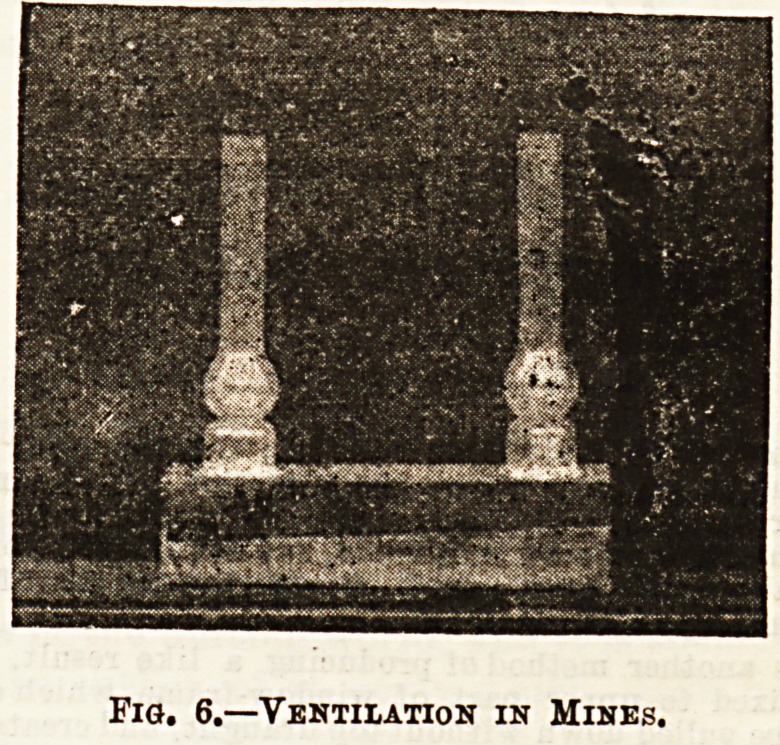


**Fig. 7. Fig. 8. f3:**
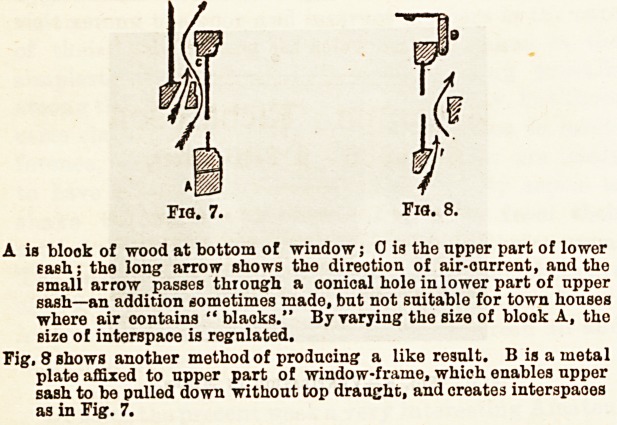


**Fig. 9. f4:**
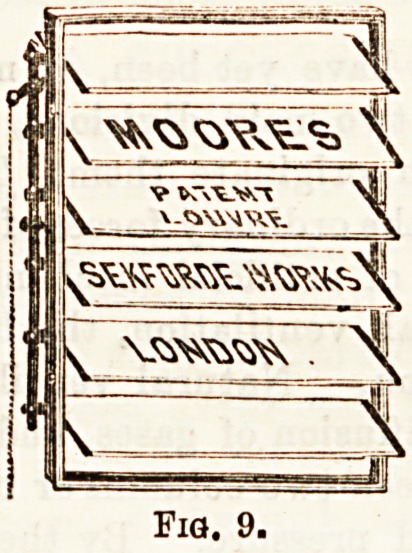


**Fig. 10. f5:**